# The mechanisms and treatments for sarcopenia: could exosomes be a perspective research strategy in the future?

**DOI:** 10.1002/jcsm.12536

**Published:** 2020-01-27

**Authors:** Shuang Rong, Liangliang Wang, Zhao Peng, Yuxiao Liao, Dan Li, Xuefeng Yang, Andreas K. Nuessler, Liegang Liu, Wei Bao, Wei Yang

**Affiliations:** ^1^ Department of Nutrition and Food Hygiene, Hubei Key Laboratory of Food Nutrition and Safety, Tongji Medical College Huazhong University of Science and Technology Wuhan China; ^2^ Department of Nutrition and Food Hygiene, School of Public Health, Medical College Wuhan University of Science and Technology Wuhan China; ^3^ Department of Nutrition and Food Hygiene and MOE Key Lab of Environment and Health, School of Public Health, Tongji Medical College Huazhong University of Science and Technology Wuhan China; ^4^ Department of Traumatology, BG Trauma Center University of Tübingen Tübingen Germany; ^5^ Department of Epidemology, College of Public Health University of Iowa IA USA

**Keywords:** Sarcopenia, Muscle loss, Aging, Exosomes, Dietary strategies, Research direction

## Abstract

The age‐related loss of muscle mass and muscle function known as sarcopenia is a primary contributor to the problems faced by the old people. Sarcopenia has been a major public health problem with high prevalence in many countries. The related underlying molecular mechanisms of sarcopenia are not completely understood. This review is focused on the potential mechanisms and current research strategies for sarcopenia with the aim of facilitating the recognition and treatment of age‐related sarcopenia. Previous studies suggested that protein synthesis and degradation, autophagy, impaired satellite cell activation, mitochondria dysfunction, and other factors associated with muscle weakness and muscle degeneration may be potential molecular pathophysiology of sarcopenia. Importantly, we also prospectively highlight that exosomes (small vesicles) as carriers can regulate muscle regeneration and protein synthesis according to recent researches. Dietary strategies and exercise represent the interventions that can also alleviate the progression of sarcopenia. At last, building on recent studies pointing to exosomes with the roles in increasing muscle regeneration, mediating the beneficial effects of exercise, and serving as messengers of intercellular communication and as carriers for research strategies of many diseases, we propose that exosomes could be a potential research direction or strategies of sarcopenia in the future.

## Introduction

Aging of the population is an increasing problem in both developed and developing countries.^1^ According to a global aging analysis reported by the World Health Organization in 2011, the world's 65‐and‐over population would triple in 2050 compared with the assessed 524 million in 2010, with the most rapidly aging populations expected in developing countries. The older population of India and China is forecasted to exceed 227 million and 330 million by 2050 from 60 million and 110 million in 2010, respectively.^2^ Aging is the very notable risk factor for many diseases^3^ such as heart disease, stroke, diabetes, and cancer, affecting health care and social costs.^2^

**Figure 1:**
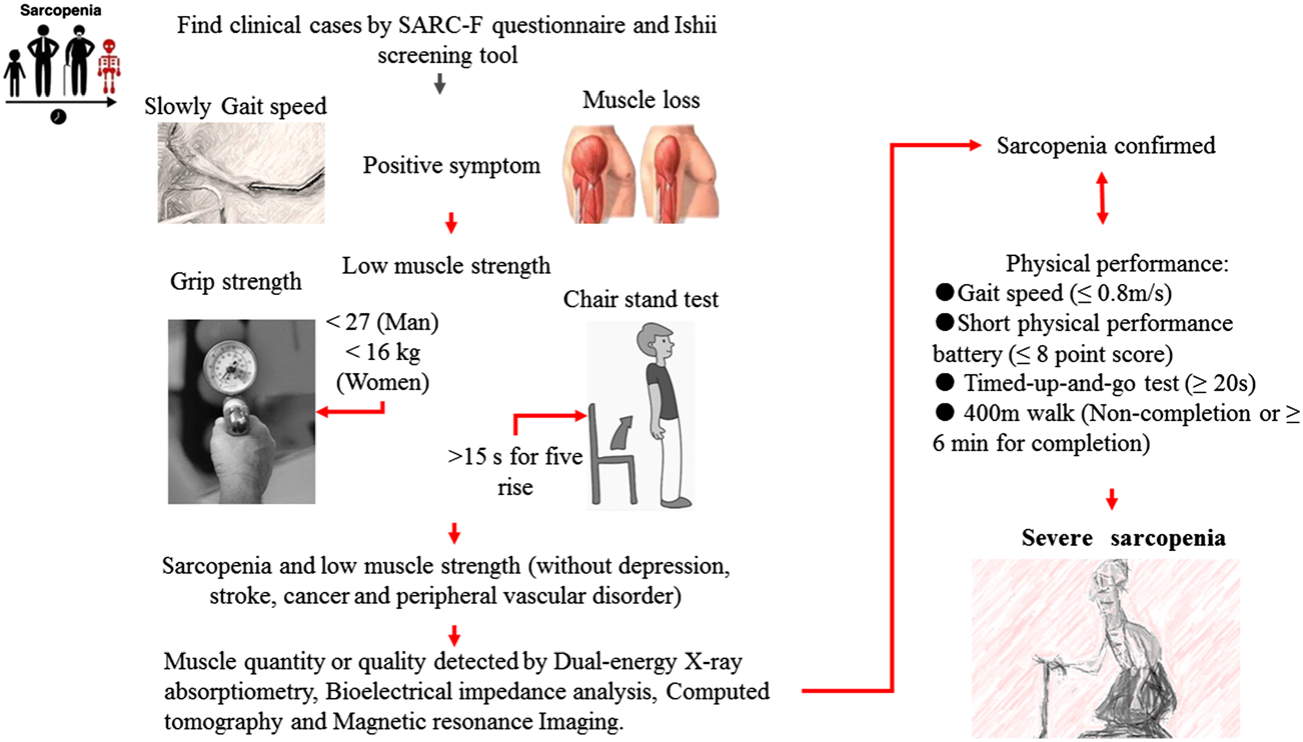
Diagnostic criteria of sarcopenia. *Note*: This picture briefly demonstrates critical diagnostic criteria for sarcopenia, some approaches for sarcopenia case finding, and measurements of muscle in clinical practice and research that also need to follow or consider the latest guideline.^12^, ^17^

Sarcopenia is one of the hallmarks of the aging process,^4^, ^5^ causing many daily life problems faced by the elderly.^6^ In 2016, a new *International Classification of Diseases, Tenth Revision, Clinical Modification* (M62.84) code was introduced for sarcopenia, stating sarcopenia as a disease.^7^ In 2010, the European Working Group on Sarcopenia in Older People (EWGSOP) introduced the first and now widely used definition for diagnosing and assessing sarcopenia.^8^ Other working groups developed similar definitions for sarcopenia as well, adding further associated aspects.^9^, ^10^, ^11^ In 2018, the European Working Group on Sarcopenia in Older People 2 (EWGSOP2) updated the definition and a diagnostic guideline of sarcopenia, associating it with low muscle strength, muscle quantity/quality, and physical performance, of which low muscle strength is accepted as a primary characteristic of sarcopenia (*Figure* *1*).^12^ The Asian Working Group for Sarcopenia (AWGS) also published regional consensus guidelines appropriate for Asian populations in 2014^13^ and updated them in 2016.^14^

In these guidelines, the muscle strength can be measured by either grip strength or the chair stand test, and muscle mass can be measured by dual‐energy X‐ray absorptiometry (DXA), magnetic resonance imaging (MRI), or computed tomography (CT). Calf circumference measures have been shown to predict performance and survival of older adults^15^ and thus may be used as a diagnostic proxy when no other muscle mass diagnostic methods are available. Beyond that, gait speed, the short physical performance battery, the Timed Up and Go test, or the 400 m walk can be also used for the assessment of physical performance. Besides, DXA is highly acceptable technique in preliminary screening for body composition assessment with the advantages of low radiation exposure and costs.^16^, ^17^ However, the availability and use of DXA for the measure of skeletal muscle mass are not very broad in clinical practice by Belgian and Latin American geriatricians.^18^ In terms of the evaluation of muscle density and assessing cross‐sectional areas (CSAs) of human muscles, CT and MRI are considered as ‘gold standard’ techniques,^16^ but they are both of higher technical complexity and costs.^16^, ^19^ Emerging literatures highlighted that ultrasound may be also useful at the bedside for assessment of muscle or lean tissue mass.^20^, ^21^ Ultrasound‐derived rectus femoris CSA appears to be a reliable index of total quadriceps volume as a simple measure of muscle size in non‐dialysis chronic kidney disease patients.^22^ In the future, bio‐impedance analysis, ultrasound, creatine dilution techniques,^23^ or specific biomarkers may be as good or more accurate approaches for estimating muscle mass (*Figure* *1*). Therefore, it is necessary to develop easily applicable and affordable techniques in the future.

Human muscle undergoes constant changes with the most alterations taking place with age. As shown by different studies, on average, the prevalence of sarcopenia in older adults aged 60–70 years lies at 5–13%, increasing to 11–50% in people aged 80 or older.^24^ Sarcopenia is closely related to negative outcomes in older adults,^16^ such as an increased risk of falls and fractures^25^ and impaired cognitive function and physical performance.^26^, ^27^ These factors further lead to the negative clinical outcomes and socio‐economic consequences, such as poor quality of life and higher overall health care cost.^28^ Researchers estimated that the economic costs for sarcopenia in the USA were about $18.5bn in 2000. Strikingly, if the prevalence of sarcopenia would be reduced by only 10%, it would save $1.1bn in medical costs per year in the US health care expenditures.^29^ Therefore, it is urgent to develop more effective research strategies and therapeutic approaches for preventing sarcopenia based on a better understanding of the potential mechanisms of this disease.

**Figure 2:**
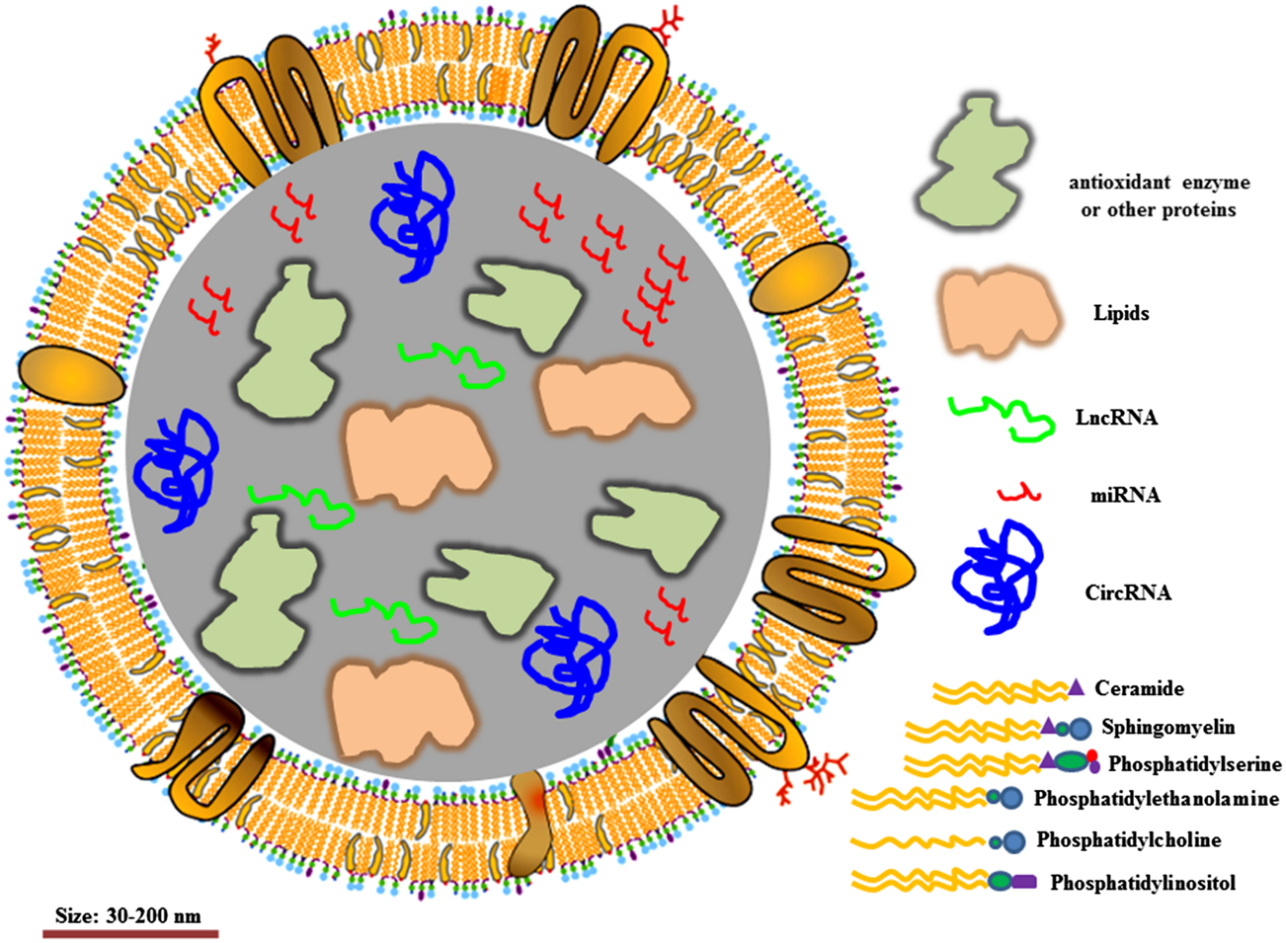
Structure of exosome. *Note*: Exosomes contain different lipids, miRNA, LncRNA, circRNA, and enzymes.

Over the years, many researches have investigated physiological and pathological conditions related to poor muscle regeneration in sarcopenia. However, the underlying molecular mechanisms associated with sarcopenia remain not completely understood. Some evidence suggests that such factors as anabolic resistance and endothelial dysfunction may contribute to the development of sarcopenia.^30^ Another one of the recent studies reported that exosomes released by muscles into the bloodstream may also play an essential role in the muscle regeneration.^31^ This opens a novel field of research in preventing muscle loss. It is hypothesized that exosomes as carrier of a cargo of proteins, mRNA, miRNA, and other non‐coding RNAs (*Figure* *2*) play a crucial role in myogenesis and muscle development. It has been shown in a mouse model of muscle injury that human skeletal myoblasts (HSkM)‐derived exosomes containing all sorts of signal molecules can promote muscle regeneration.^31^ In addition, whey protein‐derived exosomes may enhance muscle protein synthesis and hypertrophy as well.^32^

Although the treatment of sarcopenia remains challenging, it is widely accepted that such strategies as nutritional supplementation^33^, ^34^ and physical training (both aerobic exercise and resistance exercise)^35^, ^36^, ^37^ are the key interventions that can help maintain skeletal muscle mass. However, the molecular mechanisms behind the prevention from the age‐related muscle loss by nutrition and exercise are still poorly understood. More recent data indicate that exercise attenuates sarcopenia mainly through increasing peroxisome proliferator‐activated receptor gamma coactivator 1 alpha (PGC1α) signalling,^38^ which can be activated by heat shock protein (Hsp)60‐bearing exosomes released after physical training.^39^ This connection between sports and release of exosomes supports the assumption about the key role of exosomes in muscle regeneration. Furthermore, emerging evidence shows that exosomes may lay a foundation for the research strategies for many diseases, including type 2 diabetes (T2D),^40^, ^41^ age‐related bone loss and osteoarthritis,^42^ hindlimb ischaemia,^43^ myocardial infarction,^44^ Parkinson's disease (PD),^45^ and cancer.^46^ In this context, specific circulating miRNA or other modulators included in exosomes may be useful biomarkers for the diagnoses and management of disease.^46^, ^47^ Likewise, exosomes could be potentially used as drug delivery vehicles in clinical field for research strategies of different diseases, in particular those of musculoskeletal function. Alongside with the reduction in the production costs and lesser complexity of drug delivery vehicles, the advantage of safety is one of the most important issues to be mentioned.^42^, ^45^, ^46^

The aim of the review is to present and to discuss the molecular mechanisms related to the pathophysiology of sarcopenia. Besides, we will explore the role of such interventions as nutrition and sports in sarcopenia. A special focus will be laid on exosomes, which may present new effective strategies for the further research of sarcopenia. We hope that this review will provide essential information in general attempt to understand the sarcopenia and, thus, facilitate research and therapeutic strategies in the future.

## Epidemiologic studies for sarcopenia

Sarcopenia has become a global health problem.^48^ Muscle mass declines by 1–2% per year in persons older than 50 years, while muscle strength decreases at rate of 1.5% between 50 and 60 years old, and 3% thereafter.^37^ The evidence indicated that both low muscle mass and function seemed to play a synergetic role in losing physical independence.^49^ Many studies were conducted to evaluate prevalence of sarcopenia in different countries. According to a cross‐sectional population survey with 1291 subjects, the overall sarcopenia prevalence was 13.9% among community‐dwelling elderly in Pelotas, Brazil, whereas approximately one in ten elderly people aged 60–69 years was in the preclinical phase of sarcopenia.^50^ The prevalence of sarcopenia among elderly Asian was estimated at ~10%.^14^ According to one recent Korean study, where height‐adjusted and weight‐adjusted definitions were applied to diagnose sarcopenia and sarcopenic obesity, the prevalence of sarcopenia in the Korean older population was estimated at 12.4% and 9.7% in men and 0.1% and 11.8% in women, respectively.^51^ A prospective study on European men based on three different definitions of EWGSOP, International Working Group on Sarcopenia (IWGS), and Baumgartner showed that the incidence of sarcopenia was 1.6%, 3.0%, and 8.1%, respectively.^52^ Another study evaluated the data from 3025 non‐disabled elderly women by applying six different definitions of sarcopenia. The analysis demonstrated that the estimated prevalence of sarcopenia was in the wide range of 3.3–20.0%. According to all definitions considered together, only 3.1% participants could be diagnosed with sarcopenia.^53^ Therefore, it is important to continuously update definition of sarcopenia to adapt it to the scientific findings and clinical evidence with the aim to reach the conformity of clinical diagnoses and better treatment of people with sarcopenia.^12^, ^14^

## Molecular mechanisms of sarcopenia

### Recent studies for physiology of skeletal muscle

Skeletal muscle, accounting for ~40% of adult human body mass,^54^, ^55^ is composed of myofibres (also called multinucleated cells),^54^ neurons, connective tissues, and vasculature networks, of which myofibres have a primary role in structure and function of skeletal muscle.^55^ There are four major fibre types in mammalian muscles: one slow type (type I) and three fast types (types IIa, IIb, and IIx/d).^56^ During the formation and repair process, myofibres are developed through fusion of myoblasts^55^ supplied by satellite cells.^57^ Satellite cells, a population of muscle stem cells with the ability to self‐renew, play an indispensable role in muscle regeneration, which begins with muscle fibre necrosis and ends with new myofibre formation.^55^ A recent study identified a new mechanism for muscle growth and regeneration in rodents associated with Twist2‐expressing myogenic progenitors, a previously unrecognized stem cell population that differs from Pax7+ satellite cells. These Twist2+ progenitors are especially linked to type IIb/x fibres and can be seen as the first example of a fibre type‐specific myogenic progenitor population.^56^

Transmembrane proteins are other important factors in myofibre formation during muscle development. Myomixer, a new muscle‐specific membrane micropeptide in cooperation with myomaker (also called Tmem8c), can induce both fibroblast–myoblast and fibroblast–fibroblast fusion.^54^, ^58^, ^59^, ^60^ Although a large number of proteins have been demonstrated to take part in myoblast fusion, the molecular basis of myoblast fusion has not been fully understood.^61^ In their study on specific function of myomaker and its pathways, Gamage *et al*.^62^ confirmed that myomaker functioned at the plasma membrane of myoblasts to drive fusion. This was possibly regulated by appropriate myomaker localization and trafficking related to palmitoylation of C‐terminal cysteine residues and C‐terminal leucine.^62^ A previous study has provided further understanding of the molecular basis of myoblast fusion by investigating myomixer in zebrafish, turtle, and elephant shark and demonstrated the necessity of myomixer for muscle formation. A striking functional conservation of myomixer in these species was observed as well, whereas the turtle myomixer seems to be the shortest micropeptide with the ability to induce cell fusion.^61^ Therefore, these results from the newest studies probably could be investigated relationship or expression pattern in sarcopenia population such as Twist2+ progenitors and myomixer in the future.

### Current views of pathophysiological changes in sarcopenia

#### Protein synthesis and degradation

Sarcopenia is always associated with the age‐related decrease in muscle mass and function.^9^ The loss of muscle mass, which may precede gradual diminution in muscle function, takes place when the rate of muscle protein breakdown exceeds the rate of protein synthesis, thus leading to a net negative balance.^11^ The reduced sensitivity of muscle protein synthetic to dietary protein, also called ‘anabolic resistance’, is the main potential mechanism of the gradual loss of muscle mass during aging.^11^ Two of the characteristic mammalian amino acid sensing pathways, the mammalian target of rapamycin complex 1 (mTORC1)^30^, ^63^ and activating transcription factor 4 (ATF4),^30^ may have crucial roles in skeletal muscle aging. MTORC1 is known to be the major effector of food‐induced activation of skeletal muscle protein synthesis in terms of protein metabolism. However, its activation in aged muscle appears to be impaired by reduced sensitivity of protein synthesis to amino acids, while the phosphorylation state of some components of the signalling pathway seems higher in the basal state.^64^ Anabolic resistance in aged skeletal muscle may be induced by the endothelial dysfunction as well as impaired amino acid delivery, which results in muscle weakness and atrophy through decreasing mTORC1 activity and increasing ATF4 activity.^30^ Muscle atrophy occurs as a result of different pathological states and is mainly caused by accelerated protein degradation, which is mediated by muscle atrophy F‐box protein (MAFbx)/atrogin‐1 as well as muscle RING‐finger 1 (MuRF1), both muscle‐specific ubiquitin ligases.^65^ Alternatively, specific regulating molecules related to muscle wasting are either activated by factors such as the ubiquitin–proteasome system (UPS) or inhibited by insulin‐like growth factor 1 (IGF‐1) and others. IGF‐1 is known to increase protein synthesis, decrease protein degradation, and, hence, antagonize muscle wasting by activating the phosphoinositide 3‐kinase/Akt‐protein kinase B/mammalian target of rapamycin (mTOR) (PI3K/Akt/mTOR) pathway.^38^

According to another study, impaired transport of amino acids, in particular branched‐chain amino acids, into muscle had a negative effect on protein recycling and energy utilization, which was associated with alterations in phospholipid metabolism.^66^ It is also reported that hyperphosphataemia induces senescence of cultured myoblasts by impairing autophagy and activating mTOR through integrin‐linked kinase overexpression, which could be another potential mechanism of sarcopenia.^67^

#### Autophagy

According to recent studies, the detrimental effects of aging on skeletal muscle homeostasis go along with the collapse of the proteostasis network caused by dysregulation in the autophagy–lysosomal system and the UPS, both closely associated with the removal of misfolded proteins and damaged organelles. Changes of muscle proteostasis during aging are related to the affected redox regulation as well.^68^ As the removal of the damaged tissue is essential for proper muscle regeneration, the autophagy becomes a new scientific target. Autophagy, a process of cellular catabolism, becomes more important as a key regulator of muscle regeneration, influencing stem cell and myofibre function.^69^ There are three main autophagy pathways including macroautophagy, chaperone‐mediated autophagy, and microautophagy, of which macroautophagy is associated with recycling proteins and cytoplasmic organelles.^70^, ^71^ Recent works proved autophagy to be deficient in aged muscle stem cells,^70^ which can lead to increase in reactive oxygen species (ROS) levels,^72^ can reduce regenerative capacity in aged satellite cells,^70^ and can drive senescence.^72^ Age‐associated decreased macroautophagy in myogenic factor 5‐positive (Myf5+) progenitors might contribute to metabolic defects and sarcopenia.^73^

Sakuma *et al*.^74^ have explored the pathway of autophagy signalling in sarcopenic muscle of mice, evaluating the change of the amounts of the sequestosome 1 (p62/SQSTM1), LC3, and Beclin‐1 in aged muscle. A significant increase in the amount of some autophagy‐linked molecules, such as p62/SQSTM1 and Beclin‐1, was detected in mice muscle, which might be indicative of an autophagic defect. LC3‐I protein content did not increase significantly, and its active form (LC3‐II) was not detected.^74^ This leads to the conclusion that sarcopenia in mice might involve a significant defect of autophagy signalling. However, another study showed the up‐regulation of LC3‐II in a mouse model of sarcopenia.^75^

In addition, the involvement of AMP‐activated protein kinase (AMPK) in autophagy system should be considered in regard to as sarcopenia as well. AMPK could promote autophagy in skeletal muscle cells through activation of FoxO3a and interplay with unc‐51‐like kinase 1.^76^ Moreover, White *et al*.^77^ observed a reduction in autophagy in skeletal muscle stem cells with aging, which may be due to the inactivation of the AMPK/p27^Kip1^ pathway.^77^

#### Mitochondrial dysfunction

Rising number of evidence supports the key role of mitochondria in the process of sarcopenia.^35^, ^78^ Mitochondria, which provides energy for normal function of cells, are central regulators in skeletal muscle and, thus, crucial in aging process.^79^ A recent study demonstrated that mitochondrial proteostasis and fission are altered in the aged muscle. Fission and mitophagy are proved to be increasing with aging with a simultaneous decrease in mitochondrial content in both slow and fast muscle fibre types.^80^ It is well known that mitochondria play a considerable role in bioenergetics and metabolism.^3^ The accumulation of oxidative damage to macromolecules, such as mitochondrial DNA, RNA, and proteins, is supposed to contribute to mitochondrial dysfunction.^48^ Mitochondrial dysfunction includes different features, such as changed mitochondrial morphology and reduced mitochondrial content as well as reduced activity of the complexes of the electron transport chain. Other features, like opening of the mitochondrial permeability transition pore, contributes to apoptotic death of skeletal muscle cells, whereas increased ROS formation leads to mitochondrial damage.^3^

As apoptosis is correlated with energy level of cell, it may occur owing to the decreasing mitochondrial function and energy deficit within the cell in aged skeletal muscle.^48^ Kob *et al*.^81^ found that caspase‐3‐dependent apoptosis induction seemed to be the key determinant of the age‐related muscle loss in male rats.^81^ It is accepted that mitochondrial synthesis is associated with the activation of the PGC‐1α–NRF1–TFAM pathway, which is impaired in aging. This leads to a decline in both mitochondrial function and content (mitochondrial deficiency) and consequently contributes to the development of sarcopenia.^79^ It has been also hypothesized that the reduced ability of mitochondria to produce energy in muscle cells during aging can be the main driver of sarcopenia.^66^ However, considering the complexity of the factors, playing a role in mitochondrial dysfunction and their effects on aging of skeletal muscle, there are numerous diverse mechanisms underlying sarcopenia that remain to be fully elucidated.^3^

## Current preventive strategies for sarcopenia

The treatment of sarcopenia remains challenging, but there are many preventive strategies aimed at improving muscle mass and muscle function in older people, and some of them have obtained promising results.^9^ There are multiple factors that play a role in the decline of skeletal muscle performance. Besides biological aspects, such as gene^9^ and endothelial dysfunction,^82^ lifestyle aspects, such as nutritional status,^82^ physical activity,^1^ cigarette smoking,^83^ and psychosocial factors, should be taken into consideration^84^ (*Table* 1). Among all the factors, diet and physical activity may be the most effective targets for the prevention of sarcopenia during the aging process.^9^, ^85^, ^86^ The intervention strategies for sarcopenia are summarized in *Table* 2.

**Table 1:** Factors associated with muscle loss or sarcopenia

References	Biological factors	Lifestyle factors	Psychosocial factors	Other factors
Yoo *et al*.^82^	Metabolism; insulin resistance; hormone deficiency; inflammation;gene	Poor nutrition; lack of exercise; cigarette smoking		
Kim *et al*.^51^	Low IGF‐1			
Tyrovolas *et al*.^1^		Low levels of physical activity		Lower levels of wealth; higher %BF
Curtis *et al*.^83^	Hormones: plasma testosterone; growth hormone; IGF‐1; oestrogen	Nutritional status: anorexia	Self‐efficacy; psychological resiliency	
	Inflammation	Exercise		
	Insulin resistance			
	Genetics			
Kob *et al*.^81^	Endothelial function			

% BF, percent body fat; IGF‐1, insulin‐like growth factor 1.

**Table 2:** Strategies for preventing muscle mass loss or sarcopenia

References	Design origin	Sample size (*n*)	Age (year) Mean ± SD	Duration	Strategies	Main outcomes
English*et al*.^33^	Review NS	NS	NS	NS	Protein intake; exercise	Sarcopenia recommendations: the total protein intake should be 1 to 1.5 g/kg/day; doses of 50 000 IU of vitamin D a week are safe; resistance and aerobic exercise for 20 to 30 min, 3 times a week
Evans *et al*.^23^	Review NS	NS	NS	NS	Resistance training; testosterone; estrogens; growth hormone; vitamin D; angiotensin converting enzyme inhibitors; high‐caloric nutritional supplements; essential amino acids	Exercise and physical activity are important considerations for both sarcopenia prevention and sarcopenia management; nutritional interventions support muscle fibre synthesis
Scott *et al*.^9^	Review NS	NS	NS	NS	Physical activity; nutrient intake; sun exposure	Physical activity, nutrient intake, and sun exposure may have important but differing benefits for the prevention of sarcopenia in older adults
Timmerman *et al*.^100^	RCT America	Exercise group: 16 Comparison group: 4	Exercise group: 50 ± 8 Comparison group: 21 ± 2	6 weeks	Aerobic exercise	Aerobic exercise may stimulate long‐term synthesis rates of skeletal muscle DNA in older humans
Joanisse *et al*.^99^	A randomized crossover design	6	70 ± 3	NS	Aerobic exercise	Aerobic exercise can increase the anabolic response to amino acids and carbohydrate intake in healthy older adults, suggesting aerobic exercise may help prevent and treat muscle loss with aging
Booth *et al*.^85^	A systematic review	1287	65–85 (average age)	≥8 weeks	Nutritional supplementation; physical exercise	Nutritional supplementation and physical exercise are effective tools for the treatment of sarcopenia in old age
Morley *et al*.^34^	Study 1: cross‐sectional study; Study 2: case–control study; Study 3: interventional study Switzerland	80	66.6 ± 0.5	16 weeks (Study 3)	Aerobic training	Aerobic training may meliorate the loss in skeletal muscle mitochondrial content and prevent sarcopenia as well as insulin resistance
Moore^11^	Review NS	NS	NS	NS	Dietary strategies: greater meal protein intakes; leucine‐enriched protein sources. Physical activity	Physical activity should be viewed as a vital tool to help maintain nutrient sensitivity in older muscle and enhance musculoskeletal health with age
Robinson *et al*.^101^	RCT America	Young: 23 Elderly: 20	Young: 18–30 Elderly: ≥65	8 weeks	Endurance training	Endurance training can enhance muscle and mitochondrial antioxidant capacity in older participants
Van Norren *et al*.^95^	Observational and retrospective study France	146	53 ± 9	3 weeks	Caloric restriction; aerobic exercise	Caloric restriction and aerobic exercise may significantly decrease fat mass and improve lipid‐lipoprotein profile in sarcopenic obese women but does not deteriorate their sarcopenic status.
Janssen *et al*.^29^	Review NS	NS	NS	NS	Physical activity; dietary protein and leucine; pharmacologic vasodilators; ursolic acid and tomatidine	Strategies to restore amino acid delivery may attenuate sarcopenia mainly through stimulating mTORC1 and/or inhibiting ATF4 in aged muscle
Tyrovolas *et al*.^1^	Cross‐sectional study Around the world	18 363	≥65	NS	Physical activity promotion; obesity prevention	Physical activity promotion and obesity prevention are key factors for the prevention of sarcopenia syndrome
Broskey *et al*.^35^	RCT Canada	14	71 ± 5	2 weeks	Low‐load resistance exercise	Low‐load resistance exercise during short‐term inactivity is associated with greater muscle fibre area, satellite cell content, and capillarization in older men, suggesting that it is an effective countermeasure to inactivity‐induced alterations in muscle morphology with age

ATF4, activating transcription factor 4; mTORC1, mammalian/mechanistic target of rapamycin complex 1; NS, not specified.

### Dietary strategies

To maintain or restore muscle mass is indispensable for healthy aging.^64^ Nutritional supplementation can be seen as an effective tool for attenuating the process of muscle loss in sarcopenia in older people.^11^, ^86^ Adequate protein intake, such as leucine‐enriched amino acids, can improve the regeneration of skeletal muscles.^34^, ^87^ Besides, calorie restriction may represent potential means in ameliorating sarcopenic status.

#### Branched‐chain amino acid supplementation

Branched‐chain amino acids have been illustrated to preserve the body weight and cardiac function and prolong survival in rats with heart failure, probably through increasing the expression of genes involved in mitochondrial biogenesis and function in skeletal muscles.^88^ BCAA supplementation can reverse the impaired mTOR1 signalling and can increase the ability of autophagy in skeletal muscle of patients with alcoholic cirrhosis.^89^ In addition, it has the significant effects on improving glucose sensitivity and decreasing fat accumulation in skeletal muscle.^90^

Leucine is a well‐known branched‐chain amino acid, and there are many studies conducted to explore its role of in protein synthesis in the recent years. It is known that through the activation of mTORC1, leucine can promote protein synthesis, acting as a signalling molecule straight at the muscle level.^64^, ^91^, ^92^ In combination of the PI3K/Akt/mTOR pathway and UPS, leucine supplementation was shown to improve skeletal muscle regeneration in old rats through the preservation of some biological responses, such as higher number of proliferated satellite cells and bigger size of regenerating muscle fibres.^87^ Another study reported that dietary leucine may play a role in gene expression by stimulating the translation programme that involved increased energy metabolism and protein synthesis capacity.^91^

Leucine supplementation may have a partial protective effect on muscle health during relatively short periods of physical inactivity up to 14 days.^33^ However, meal‐based responses to dietary protein will decline with age or partial physical inactivity. Although present dietary recommendations for protein are 0.8 g/kg body weight of daily intake, new studies suggest that total daily coverage of protein for older people should be >1.0 g/kg body weight.^92^ Based on rat studies, where leucine supplement promoted muscle protein synthesis,^91^ a study was undertaken to evaluate the response of older human skeletal muscle to leucine top‐ups. The results showed that oral leucine, applied 90 min after adequate doses of essential amino acid feeding, had no effect on neither muscle anabolic signalling nor protein synthesis. Considering this the insusceptibility of human skeletal muscle to the anabolic effects of leucine in the postprandial period in older men, it was suggested that the leucine supplements should be taken outside of meals.^93^ Although quantitative and qualitative manipulations of leucine supply can alleviate anabolic resistance, this issue remains controversial in terms of possible adverse side effects of high‐dose, long‐term leucine supplementation on insulin resistance and tumorigenesis.^64^

#### Calorie restriction

The majority of recent studies confirm that moderate calorie restriction with appropriate nutrition not only has a protective effect against hypertension, obesity, and cardiovascular disease in human but may recover the skeletal muscle transcriptional profile as well.^94^ Data from a previous study indicated that the mTOR pathway in the caloric‐restricted aging mice might be more active than that in the control group.^95^ Sarcopenic obese individuals build a special population group that combines sarcopenia and obesity, which might need a special treatment, different from non‐sarcopenic obese individuals. Barbat‐Artigas *et al*.^96^ suggested that a mixed weight loss programme, combining caloric restriction and aerobic exercise, for both groups played possibly a role in reducing fat mass and ameliorating lipid‐lipoprotein profile in obese women but did not deteriorate the sarcopenic status. However, the loss of a lean mass was less in sarcopenic obese group, which could be explained by very short time‐span of the programme (3 weeks), higher proteins (20–25%) provided in this study than recommended by the French Agency for Food Safety for sedentary people (15%), and less muscle to lose in sarcopenic obese women compared with non‐sarcopenic obese ones.^96^

### Exercise

As the lack of sufficient activity on daily basis can be seen as a main cause of chronic diseases, it has been examined as an effective target for the prevention of chronic conditions, such as sarcopenia,^97^ metabolic syndrome, insulin resistance, cancers, and obesity.^85^ Physical activity or exercise is a promising strategy to decelerate sarcopenic process.^98^ Various types of exercise training are shown to increase capillarization in older people and likely be beneficial in ameliorating the stem cell response, ultimately reducing and/or delaying the impact of sarcopenia.^99^

#### Aerobic exercise

Aerobic exercise may support the amelioration of the loss of mitochondrial content in skeletal muscle and preventing aging muscle co‐morbidities (e.g. sarcopenia and insulin resistance).^35^ A moderately intense bout of aerobic exercise was shown to increase the subsequent anabolic response to amino acids and carbohydrate intake in healthy older subjects, indicating that aerobic exercise might be essential in the prevention and treatment of sarcopenia. This positive effect on skeletal muscle protein anabolism appeared to be associated with increased microvascular perfusion and nutrient delivery, rather than with improved insulin signalling.^100^ In another study, older exercise groups showed significantly higher levels of muscle protein synthesis, phospholipid synthesis, and DNA than did sedentary young subjects during the 6 week aerobic training. According to the authors, the aerobic exercise might stimulate long‐term synthesis rates of skeletal muscle DNA, which could be attributed to satellite cell activation rather than to mitochondrial DNA. Further studies should consider more the interrelationship between the aerobic exercise and satellite cell recruitment as well as skeletal muscle function in aging.^101^ Recently, Johnson *et al*.^102^ observed different impact of endurance training on protein quality in sedentary young and older adults. Following 8 weeks of endurance training, young adults showed a reduction in the oxidative damage to skeletal muscle and mitochondrial proteins, whereas the older individuals did not. However, older adults demonstrated increased skeletal muscle antioxidants catalase and superoxide dismutase‐2 that involved muscle antioxidant capacity.^102^

#### Resistance exercise

Resistance exercise as well can be seen as promising approach in preventing sarcopenia,^103^ which seemed to be beneficial for muscle anabolism by enhancing the efficiency of essential amino use.^92^ In 2018, Moore *et al*.^36^ explored the impact of low‐load resistance exercise (LLRE) on skeletal muscle in older people, who at the same time underwent short‐term inactivity (step reduction). They demonstrated that LLRE was associated with greater muscle fibre CSA, capillarization, and satellite cell content (PAX7+ cells) in skeletal muscle of older adults, indicating LLRE as a possible effective mechanism to delay loss of muscle mass due to inactivity or aging.^36^

## The exosome perspective

Attention on micro‐vesicles in general and exosomes in particular rose significantly in recent years, owing to their potential role as biomarkers, in cancer progression and metastasis as well as their application in enhancing tissue repair.^42^ In the following sections, we summarize the current evidence on the exosomes biogenesis, assay methods, their connection with exercise, and others. Owing to the role of exosomes as messengers of intercellular communication, they are approached as a potential research strategy in the treatment of various diseases, including sarcopenia.

### Exosome biogenesis and profile

Exosomes, an important group of extracellular vehicles (EVs), are membrane‐contained vesicles that carry protein, nucleic acid, and lipid cargoes released from a wide range of cell types.^40^, ^41^, ^104^, ^105^, ^106^ According to their size, membrane composition and content,^107^, ^108^ exosomes are defined as a homogenous population of endogenous vesicles with a diameter of 30–200 nm,^46^, ^107^, ^109^, ^110^, ^111^ which are derived from inward budding of the multivesicular body membrane^110^ and secreted into the outside of the cells upon fusion of multivesicular body and plasma membrane.^112^ EVs are present in various body fluids, such as blood, urine, saliva, synovial fluid, bile, cerebrospinal fluid, amniotic fluid, breast milk, seminal plasma,^104^, ^113^ and human nasal secretions.^114^ Their appearance reflects specific physiological and pathological conditions.^115^ Current research has also emphasized the role of EVs carrying selectively packaged cargo.^116^

### Technologies for analysis of exosomes

Generally, microscopy‐based methods are used techniques for observing and characterizing physical features of EVs.^117^ However, it is extremely challenging to isolate exosomes from multiple biological fluids with high purity as well as high throughput because of their intrinsic small sizes.^118^ Ultracentrifugation and ultrafiltration are conventional methods but not quite efficient in separating exosomes and other micro‐vesicles.^112^, ^119^ Recently, new developed technologies are applied to improve and achieve better separation of exosomes.^112^, ^118^, ^120^

Immunoaffinity capture is a marker‐dependent approach that allows to capture EVs with particular antibodies against EV markers, such as the tetraspanins and tumour‐associated markers.^117^ Exosomal markers include ALG‐2‐inter‐acting protein X (Alix), TSG101, CD63, CD81,^44^, ^45^ Flottilin 1,^119^ FLOT‐1, ICAM, EpCAM, ANXA5,^31^ SDCBP, CD9,^121^ and CD82,^46^, ^122^ whereas GM130 is an non‐exosomal marker.^31^ Other techniques include enzyme‐linked immunosorbent assay as well as such conventional protein analyses as mass spectrometry^113^ and western blotting.^117^

Although there are some conventional and new established techniques for the isolation of the exosomes, such as ultracentrifugation and asymmetric‐flow field‐flow fractionation, it remains extremely challenging to separate them from other micro‐vesicles with high purity and high throughput. Further study is needed in the future to improve techniques of separating exosomes.

### Exosomes as messengers of intercellular communication

EVs (in particular exosomes) participate in communication between surrounding or distant cells, delivering signals across tissues and organs^110^, ^115^, ^123^ through carrying cytoplasmic and membrane proteins, such as receptors and major histocompatibility complex molecules,^110^ growth factors, cytokines, nucleotides, and metabolites as well as miRNAs.^124^ Thus, exosomes play a key role in various biochemical processes.^40^, ^115^, ^123^

The essential role of exosomes in organ crosstalk highlights the necessity for future efforts in this area.^125^ Exosomes released by their mother cell can fuse with target cells and exchange cytosol and membrane proteins between two cell types.^37^ More studies confirmed that mRNA and miRNA can be transported by exosomes to another cell, as a means of cell–cell communication.^126^ According to the data of the previous study, miR‐23a/27a produced in muscle was delivered by circulating exosomes to kidney, where they mitigated the fibrosis process.^127^ Another study reported that muscle‐secreted factors carried within exosomes, such as proteins (myokines), metabolites, and miRNAs, influence the survival or the function of β‐cells, which can be one of underlying molecular mechanisms of T2D, and, thus, a potential new target for novel preventive strategies against T2D.^125^

### Exosomes as carriers or regulators in many diseases

In 2016, a research proposed that exosomes enriched with exerkines,^128^ a term introduced to describe all factors released by skeletal muscle in response to endurance exercise, would be future research strategies for obesity and T2D.^40^ As the maintenance and stability of transplanted exosomes *in vivo* over a longer time period are very challenging, Zhang and his colleagues examined human placenta‐derived mesenchymal stem cell‐secreted exosomes, incorporated with chitosan hydrogel, in a murine model of hindlimb ischaemia, and concluded that this strategy could enhance therapeutic function of exosomes.^43^ It is also reported that mesenchymal stem cell exosomes could mitigate temporomandibular joint osteoarthritis in a rat model by reducing inflammation and recovering matrix homeostasis.^129^ Recently, Pan *et al*.^130^ discovered that miR‐21 (an anti‐apoptotic miRNA) was carried from preischaemic limbs to distal organs via serum exosomes and demonstrated a crucial role of exosomal miR‐21 in renal protection, suggesting exosomes as the possible research strategies in response to sepsis‐induced kidney injury.^130^ Further, recent studies showed that exosomes appear to play a positive role in improving cardiomyocyte survival, highlighting the importance of non‐invasive strategies, such as exosomes, in stimulation of the endogenous cardiac repair and regenerative mechanisms.^44^, ^124^ Moreover, another study also confirmed exosomes as mediators of anti‐aging properties of cardiosphere‐derived cells in aged rats.^131^

One of the biggest challenges in treatment of PD is to deliver drugs across the blood–brain barrier.^45^ Considering that the large amount of evidence demonstrated the ability of exosomes to overcome the barriers,^132^, ^133^ it would be a logical next step to use exosomes as drug‐loaded carriers. A recent study demonstrated dopamine‐loaded exosomes successfully delivering dopamine to brain and showed higher therapeutic efficacy as well as lower systemic toxicity in a PD mouse model.^45^

Exosomes have already been utilized in cancer research as well.^109^, ^134^ To overcome such shortcomings as low drug‐loading efficiency, some studies try to develop artificial exosome constructions. A recent publication describes artificial chimeric exosomes constructed by embedding cell membrane proteins derived from red blood cells and MCF‐7 cancer cells into synthetic phospholipid bilayers, thus combining corresponding targeting capability from cancer cells and anti‐phagocytosis capability from red blood cells. Upon application, artificial chimeric exosomes had achieved higher tumour accumulation, reduced interception, and improved the antitumour therapeutic effect with low toxicity and high drug‐loading capacity.^109^ Taking into account that exosomes participate in initiation, progression, and metastasis of cancer tumours, they can be considered as potential instrument in early diagnosis of this disease, as shown in a recent study.^46^

### Exosomes as mediators in muscle of the beneficial effects after exercise

As it was shown in some studies, physical exercise, which is associated with muscle regeneration, growth, aging, and sarcopenia, can induce rapid release of exosomes into the circulation.^115^, ^121^ Exosomes were observed to be significantly increased immediately after the intensive bout of cycling and being dropped again within 90 min at rest.^115^ A research on animal model has demonstrated that exosomes released with exercise down‐regulated the levels of matrix metalloprotease 9 in the heart tissue of diabetic mice by up‐regulating miR‐29b and miR‐455, hence mitigating the deleterious downstream effects of matrix metalloprotease 9.^119^ A large number of researches demonstrated that Hsp, Hsp72 (Hsp70) in particular, is released into the human circulation together with exosomes during exercise.^115^, ^135^ Similarly, a recent paper showed that endurance training increased the release of skeletal muscle Hsp60 into the blood of mice, which were carried by exosomes, promoting the activation of PGC1α isoforms in the interstitial cells.^39^ The growing body of evidence supports the impact of exercise on increasing of PGC1α signalling, which plays a crucial role in research of sarcopenia.^38^

### Skeletal muscle regeneration: are exosomes a potential direction for the research

strategies of sarcopenia?

Skeletal muscle has an ability to regenerate itself after injury or disease through fusion of satellite cells with damaged myofibres.^58^ Evidence clearly indicates that in elderly the satellite cells are reduced in their content and function.^99^ Notably, impaired satellite cell activation was considered to be one of the possible important mechanisms of sarcopenia.^38^ As it was shown in a recent study, sarcopenia mainly affects fast muscle fibres of elderly with a lower expression of myosin, actin chaperones, and proteasome activity than in the corresponding slow fibres.^80^ However, another study demonstrated that this assumption should be handled with some caution if a severe muscle atrophy is attested.^136^ Besides, the larger distance between satellite cells and capillaries observed in elderly may lead to the dysregulation in satellite cells activation, consequently impairing muscle remodelling and, under circumstances, regenerating. Considering this, an optimal satellite cell activation parallel to the skeletal muscle capillarization can also be of high importance in order to maximize the potential regeneration of skeletal muscle in older adults.^99^

Some recent studies hypothesize that circulating microRNAs (miRNAs), small non‐coding RNAs, transiently or adaptively influenced by acute and chronic exercise, may also act as modulators of skeletal muscle function.^137^ The miRNAs are involved in post‐transcriptional gene regulation through mRNA degradation or translational inhibition,^137^ regulating ~60% of protein‐coding genes, which are associated with various biological processes.^138^ Skeletal muscle is rich in miRNAs with specific functions^137^ such as miR‐1, miR‐133,^139^ miR‐181a‐5p,^140^ miR‐206,^139^ miR‐208, miR‐486, miR‐499,^137^ miR‐31,^116^ and miR‐23a/27a.^127^ For example, the miR‐31 is involved in regulating translation of the satellite cell activator Myf5 as well as in muscle regeneration,^116^, ^141^ and miR‐23a/27a can attenuate muscle atrophy.^127^ Notably, as it is shown in some research papers, extracellular miRNAs may be secreted into EVs (e.g. exosomes), which carry them to the targeted cells.^42^ For instance, EVs‐miR‐133b and EVs‐miR‐181a‐5p were observed to be significantly up‐regulated after exercise, which possibly may be related to muscle remodelling and homeostasis.^140^ Recently, it has been discovered that muscle‐derived EVs carrying senescence‐associated miRNAs, such as miR‐34a‐5p (miR‐34a), were able to induce cellular senescence in bone stem cells.^142^ Exosomes belong to EVs and carry significant information, such as proteins, DNAs, miRNAs,^118^ and circular RNAs.^143^ The secreted exosomes deliver specific signal molecules, including myogenic growth factors, such as IGFs, fibroblast growth factor‐2, and hepatocyte growth factor .^31^

A recent study demonstrated that exosomes derived from HSkM, can trigger myogenesis of stem cells, providing important biochemical cues for muscle regeneration.^31^ In the meantime, Mobley *et al*.^32^ carried out the first study to demonstrate that whey protein‐derived exosomes increased muscle protein synthesis and hypertrophy *in vitro*. This could be associated with an unknown mechanism that increased translation initiation factors [eukaryotic initiation factor 4A (eIF4A) mRNA] without enhancing mTOR signalling or relation to bovine‐specific miRNA. Owing to the overexpression of miRNAs in bovine‐specific exosomes, the authors suggest further investigation on their influence on myotube muscle protein synthesis and anabolism.^32^

Decline in skeletal muscle regenerative capacity with age contributes to the onset of sarcopenia.^69^ Exosomes, derived from HSkM or whey protein, can enhance muscle regeneration^31^ and muscle protein synthesis.^32^ In addition, exosomes were shown to be promising direction in research strategies of such diseases as T2D,^40^ hindlimb ischaemia,^43^ temporomandibular joint osteoarthritis,^129^ sepsis‐induced kidney injury,^130^ myocardial infarction,^44^ PD,^45^ and gastric cancer^46^ (*Table* 3). As such, it can be possible to engineer exosomes that contain specific DNA, RNA, proteins, or a drug to be delivered to targeted cells, offering considerable advantages in research strategies of sarcopenia.

**Table 3:** Exosomes make as carriers or regulators in many diseases

References	Main methods	Target organs/tissues	Main results/the roles of exosomes	Conclusions
Chaturvedi *et al*.^119^	The mice (a model of T2D) were exercised for 8 weeks	Heart	Down‐regulation of MMP9 expression after exercise; increased expression of miRs (29b and 455)	Exosomes released with exercise down‐regulate the expression of MMP9 in the heart tissues by the means of miR‐29b and miR‐455, preventing the detrimental effects of MMP9
Barone *et al*.^39^	Review	Pancreas, adipose tissue, liver, skin, heart, brain and kidney	Endurance exercise can mitigate the effects of metabolic diseases such as T2D and obesity; various peptides and miRNA species (exerkines) are changed in response to endurance exercise; exosomes can promote crosstalk between organs by carrying exerkines (proteins, nucleic acids and miRNA) between cells and tissues	Exosomes enriched with exerkines could be useful for the treatment of T2D and obesity in the future
Pan *et al*.^130^	*In vivo* studies: rats were injected neonatal rat CDCs *In vitro* studies: old human heart cells or cardiomyocytes from old rats were exposed to human CDCs or CDC‐XO	Heart	*In vivo* studies: CDCs induce biological rejuvenation of the heart *In vitro* studies: CDC‐XO mediate effects of CDCs that increased telomere activation	The *in vivo* and *in vitro* findings demonstrate that exosomes mediated the anti‐senescent effects of young CDCs
Aoi *et al*.^126^	Intramuscular injection of AAV‐miR‐23a/27a in diabetic mice	Skeletal muscles and kidney	Reduce muscle wasting: increased miR‐23a and miR‐27a; increased phosphorylated Akt; attenuated FoxO1 and PTEN proteins; reduced TRIM63/MuRF1 and FBXO32/atrogin‐1; decreased myostatin mRNA protein levels; decreased phosphorylated pSMAD2/3 Reduce fibrosis lesions: decreased phosphorylated SMAD2/3; decreased alpha smooth muscle actin; decreased fibronectin and collagen Muscle–kidney crosstalk: in serum exosomes: miR‐23a and miR‐27a increased; in the kidney: miR‐23a and miR‐27a increased, but viral DNA not detected	miRNA‐23a/27a‐containing exosome might attenuate muscle atrophy and renal fibrosis through muscle–kidney crosstalk
Lewis *et al*.^124^	Review	Skeletal muscle and pancreatic β‐cells	Muscle is an endocrine organ that can secrete myokines, miRNAs, metabolites, and factors contained within exosomes; muscle‐secreted factors show potential in mediating endocrine effects in β‐cells	Muscle‐secreted factors contained within exosomes have potential to influence the function of β‐cells in health and disease (T2D)
Murphy *et al*.^42^	Injection MSC‐derived exosomes incorporated with chitosan hydrogel in the mouse hindlimb ischaemia model	Muscle of hindlimb	miR‐126 level in hydrogel‐incorporated exosomes was significantly higher than that in exosomes; chitosan hydrogel markedly reduced the degradation of proteins in exosomes; hydrogel‐incorporated exosomes significantly reduced the necrotic fibres and inflammatory cells in injured tissues.	Exosomes with an injectable hydrogel can enhance therapeutic effects for hindlimb ischaemia
Zhang *et al*.^43^	hCMPs were transplanted onto infarcted swine heart	Heart	Transplantation of hCMP results in significant improvements in left ventricular function, infarct size, myocardial wall stress, and myocardial hypertrophy; exosomes secreted by hCMPs can promote cardiac muscle proliferation, increase the angiogenic activity of endothelial cells, and protect cardiac muscles from hypoxic damage	The exosomes that originated from the hCMP appeared to have the potential to improve cardiomyocyte survival
*et al*.^44^	PD mice were treated with dopamine‐loaded blood exosomes	Brain	Brain distribution of dopamine increased more than 15‐fold by using the blood exosomes as drug carriers; Dopamine‐loaded exosomes showed better therapeutic efficacy and lower systemic toxicity than free dopamine	Blood exosomes might be power carriers for better treatment of PD
Gao *et al*.^108^	Intravenous injection of liposomes, ACEs, AREs, and AMEs in tumour‐bearing nude mice	Liver, kidney, and tumour tissue	*In vivo* biodistribution of vesicles at 24 h post‐injection: liposomes achieved a bit of tumour accumulation, but much of that accumulated in kidney and liver Compared with liposomes: AREs, AMEs, and ACEs obtained a 2.1‐fold, 2.5‐fold, and 3.4‐fold increased tumour accumulation, respectively AREs, AMEs, and ACEs showed a 31%,12%, and 36% reduced kidney accumulation, respectively AREs, AMEs, and ACEs showed a 40%,15%, and 37% reduced liver accumulation, respectively *In vivo* antitumour efficacy of ACEs: The smallest tumour volume was found in the ACE‐treated group	ACEs had achieved higher increased tumour accumulation and better antitumour therapeutic effect than did liposomes *in vivo*

AAV, adeno‐associated virus; ACEs, integrating hybrid RBCs and MCF‐7 cell membrane proteins into synthetic phospholipid bilayers; Akt, protein kinase B; AMEs, artificial MCF‐7 cell exosomes; AREs, artificial RBC exosomes; CDC, cardiosphere‐derived cell; CDC‐XO, exosomes secreted by CDCs; FBXO32, recombinant F‐box protein 32; hCMPs, human cardiac muscle patches; miRs, microRNAs; MMP9, matrix metalloprotease 9; MSC, mesenchymal stem cell; PD, Parkinson's disease; pSMAD2/3, phosphorylation of small body size and mothers against decapentaplegic homologue 2/3; PTEN, phosphatase and tensin homologue; T2D, type 2 diabetes; TRIM63, tripartite motif containing 63.

## Conclusion

The incidence of sarcopenia is often associated with the aging process,^5^, ^16^ having a negative impact on human health. Considering a relatively high level of aged population in many counties, public policies should focus on preventing clinical progression of sarcopenia^50^ by screening for sarcopenia combined with appropriate treatment when necessary for all persons who are 60 years or older.^144^

Although a series of studies have focused on exploring the complex potential pathophysiology of sarcopenia, the underlying molecular mechanisms still remain poorly understood. Collectively, the present studies suggest that protein synthesis and degradation, autophagy, mitochondria dysfunction, and impaired satellite stem cell activation are closely associated with muscle weakness and muscle degeneration, possibly being involved in molecular mechanisms of sarcopenia.

**Figure 3:**
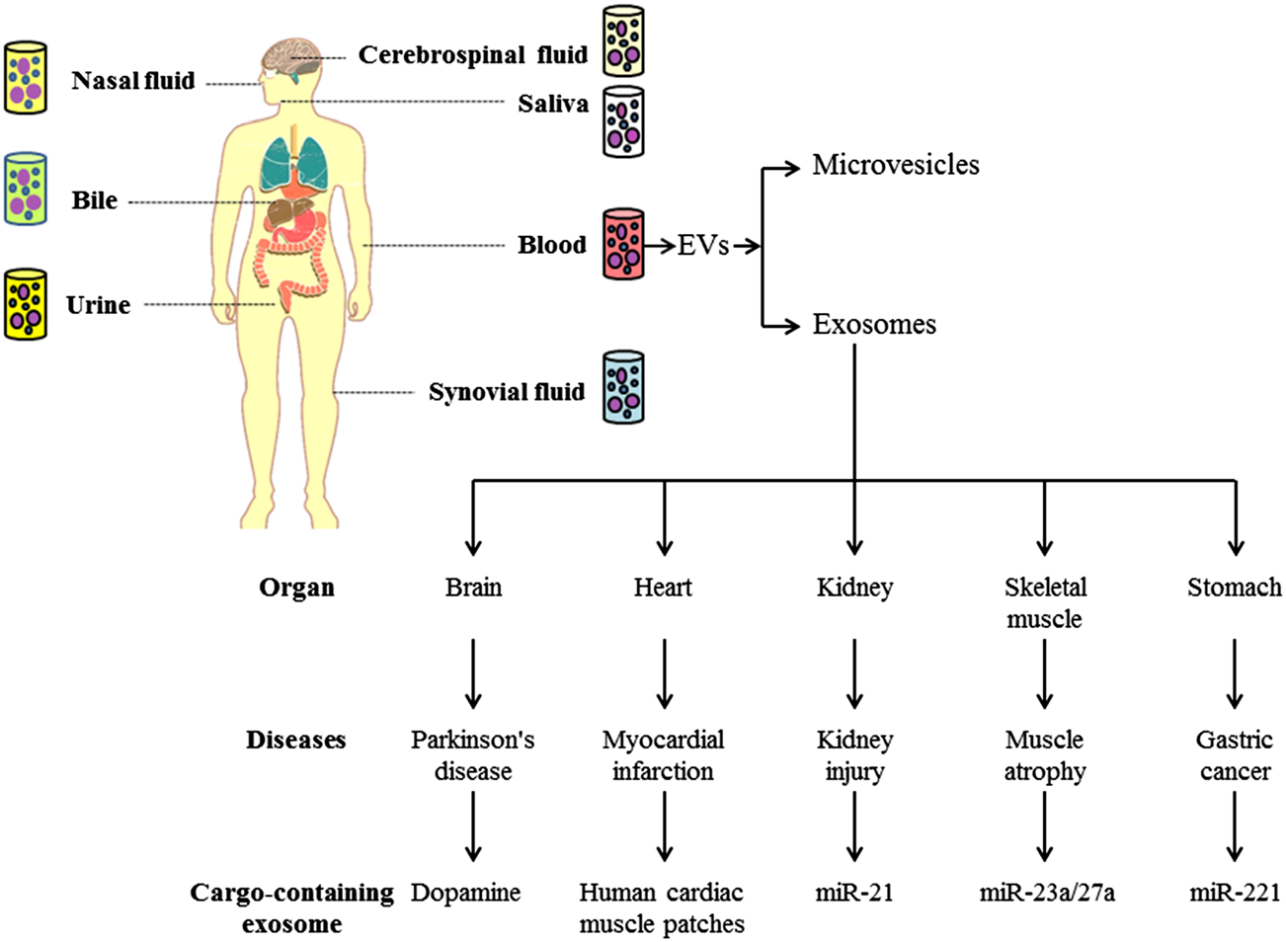
Exosomes serving as messengers for inter‐organ communication and improving diseases. *Note*: Some typical exosomes can be detected from different biological fluids. These exosomes can release different contents for regulating physiological or pathological development.

Many strategies are developed to improve sarcopenic condition in older people, of which physical activity and nutritional strategies (e.g. the leucine‐enriched protein sources) are both essential to maintain musculoskeletal health with age.^4^, ^11^, ^64^ In addition, more recent data indicate that exosomes, secreted by skeletal muscle cells and carrying miRNAs and other factors, may act as vital modulators of skeletal muscle function^65^, ^137^ and may have the potential in the research strategies of sarcopenia. Owing to their diverse pathological and therapeutic effects, exosomes has attracted more attention in the scientific community in recent years.^31^ It has been shown that exosomes are related to organ crosstalk and can be beneficial in research of many diseases, including kidney injury, myocardial infarction, PD, and cancer (*Figure* *3*). Importantly, the present data suggest that exosomes may mediate and enhance the beneficial effects of exercise.

Finally, emerging evidence has presented that exosomes as carriers can increase muscle regeneration after skeletal muscle injury and improve muscle protein synthesis and hypertrophy, which could support exosomes as vectors for future research strategies of sarcopenia. All these mechanisms are interconnected, but the underlying pathways are still not understood completely and should be examined more thoroughly in future studies.

## Conflict of interest

All the authors declare that they have no conflict of interest.
